# Current and Future Roles of Circular RNAs in Normal and Pathological Endometrium

**DOI:** 10.3389/fendo.2021.668073

**Published:** 2021-05-26

**Authors:** Jiajie Tu, Huan Yang, Yu Chen, Yu Chen, He Chen, Zhe Li, Lei Li, Yuanyuan Zhang, Xiaochun Chen, Zhiying Yu

**Affiliations:** ^1^ Department of Gynecology, Shenzhen Second People’s Hospital/The First Affiliated Hospital of Shenzhen University Health Science Center, Shenzhen, China; ^2^ Key Laboratory of Anti-Inflammatory and Immune Medicine, Ministry of Education, Anhui Collaborative Innovation Center of Anti-Inflammatory and Immune Medicine, Institute of Clinical Pharmacology, Anhui Medical University, Hefei, China; ^3^ Department of Obstetrics and Gynecology, The First Affiliated Hospital of USTC, Division of Life Sciences and Medicine, University of Science and Technology of China, Hefei, China; ^4^ The First Clinical Medical College, Southern Medical University, Guangzhou, China

**Keywords:** circRNAs, endometrium, endometriosis, endometrial cancer, high-throughput studies

## Abstract

The uterine endometrium, which lines the mammalian uterus, is essential for embryo implantation. This lining undergoes significant changes during sexual and menstrual cycles. The endometrium is also associated with hormone-related diseases such as endometriosis and endometrial cancer. Circular RNAs (circRNAs) play a role in various biological processes. Recent studies have determined that circRNAs function in both normal and pathological endometrial environments. Here, we review high-throughput studies pertaining to circRNAs as well as individual circRNAs active in the endometrium, in order to explore the myriad functions of circRNAs in the endometrium and mechanisms underlying these functions, from panoramic and individual perspectives. Owing to their abundant expression, stability, and small size, circRNAs have displayed potential usefulness as diagnostic markers and treatment targets for endometrial-related diseases. Therefore, the specific role of circRNAs in the endometrium warrants systematic investigation in the future.

## Introduction

The endometrium is a layer of cells that forms the lining of the mammalian uterus. It responds to changes in the levels of both estrogen and progesterone (1). The endometrium consists of two layers: the functional layer and the basal layer. The functional layer, which comprises a dense layer and a sponge layer, changes and is shed during the ovarian cycle. The basal layer is not affected by ovarian hormones and does not undergo periodic changes.

High-throughput sequencing has enabled the discovery of a wide range of non-coding RNAs, including microRNAs (miRNAs), long non-coding RNAs (lncRNAs), and circular RNAs (circRNAs), that function in various biological processes (2). In some cases, these non-coding RNAs exhibit abnormal expression patterns that may lead to multiple diseases (3). Unlike conventional linear RNAs, circRNAs are a unique class of RNAs whose 3′ and 5′ ends are covalently bonded to form a closed continuous loop that is resistant to exonuclease digestion (4). The high abundance, stability, and evolutionary conservation of circRNAs suggest that they play important regulatory roles (5). For example, as competing endogenous RNA (ceRNA), circRNAs may use miRNA response elements (MREs) to sponge miRNAs and strongly inhibit miRNA activity, resulting in the upregulation of miRNA targets, which ultimately affects cellular processes such as cell differentiation, proliferation, and apoptosis as well as other cellular functions (6). These properties make circRNAs important bioregulators of molecular mechanisms underlying various diseases, indicating that circRNAs may be useful as potential diagnostic biomarkers and therapeutic targets (5). However, studies related to the activity of circRNAs in the endometrium, including those involving endometrial development and endometrium-related diseases, such as endometrial cancer (EC) and endometriosis, are currently in the formative stages.

Investigating circRNA function in the endometrium as well as mechanisms underlying such functions may enhance our understanding of the molecular processes associated with the physiological development of the endometrium and provide new opportunities to develop more effective diagnostics and treatments for endometrium-related diseases. The current study attempted to review the properties and functions of circRNAs in the endometrium under both physiological and pathological conditions with a view toward the future of circRNA research.

## circRNAs in Endometrial Cancer

EC is a type of epithelial malignancy that occurs in endometrial tissue, often in perimenopausal and postmenopausal women (7). With nearly 200,000 new cases being diagnosed each year, EC has come to be considered as one of the most common female reproductive system tumors and the third most common gynecological malignant tumor (second only to ovarian and cervical cancer) (7).

### High-Throughput Studies of circRNAs in EC

RNA sequencing (RNA-seq) analysis has indicated that the total abundance of circRNAs in 1-2 grade type I EC is lower than that in the normal endometrium. In addition, many circRNAs embedded in the “hotspot” genes of EC were transcribed, explaining the differences in circRNA expression between normal and malignant endometria (8). Although the functional significance of these circRNAs in EC remains to be determined, it is surmised that these may serve as potential biomarkers of EC.

Study has focused on the role of circRNAs in extracellular vesicles (EVs) isolated from the sera of three grade 3 EC patients (9). EVs isolated from serum samples obtained from 3 EC patients and three age-matched healthy controls were subjected to RNA-seq, which indicated that the expression levels of 209 circRNAs were upregulated while those of 66 were downregulated. Kyoto Encyclopedia of Genes and Genomes (KEGG) and Gene Ontology (GO) analyses showed that differentially expressed circRNAs were associated with several signaling pathways, such as focal adhesion, extracellular matrix-receptor interaction, amebiasis, and regulation of the actin cytoskeleton. The expression levels of circ_0109046 and circ_0002577 were confirmed using a quantitative real-time polymerase chain reaction (qRT-PCR). CircRNAs enriched in circulating EVs may be useful as potential biomarkers of EC.

Another study, which focused on the role of circRNAs in grade 3 ECs, used circRNA sequencing (circRNA-seq) to detect circRNA expression characteristics in two grade 3 ECs and adjacent non-cancerous endometrial tissues (10). In total, the expression of 75,928 circRNAs was significantly changed (p<0.05). Upregulation of hsa_circ_0000437, hsa_circ_0001776, and hsa_circ_0009043 and downregulation of hsa_circ_0039569 and hsa_circ_0001610 was verified using qPCR. Bioinformatic methods (TargetScan and miRanda) indicated that hsa_circ_0039569 has MREs for hsa-miR-542-3p and hsa-let-let-7c-5p. Downregulation of hsa-miR-542-3p and hsa-let-let-7c-5p in grade 3 EC tissues was validated using qRT-PCR. Clinical-pathological parameters indicated that the expression level of hsa_circ_0039569 was significantly correlated with tumor differentiation. These results demonstrated the presence of many differentially expressed circRNAs between level 3 EC and adjacent non-cancerous endometrial tissues, thus providing new molecular candidates for diagnosis and clinical treatment of grade 3 EC.

Dou et al., identified 234 recurrent circRNAs in EC (11). In order to determine possible regulators, they examined the correlation between RNA-binding proteins (obtained from protein sequencing), and circRNAs. The level of the kH domain RNA binding (QKI) protein was positively correlated with 35 circRNAs. QKI induces epithelial-mesenchymal transition (EMT). Since miRNAs also play a key role in EMT, circRNAs may be used as miRNA sponges to regulate miRNA activity. Therefore, researchers explored miRNA binding sites in the 35 circRNAs that were correlated with QKI levels and identified potential binding sites for 36 miRNAs. The activity of these miRNAs was negatively correlated with QKI expression, indicating that the activity of these miRNAs may be inhibited by QKI, possibly *via* QKI-mediated expression of circRNAs. A set of known or predicted QKI regulators (miR-200c, miR-221, miR-130a, miR-130b, and miR-183) was among the miRNAs showing the strongest negative correlations with QKI. Such positive correlation between QKI and circRNAs, and negative correlation between QKI and the activity of specific miRNAs suggested that a novel mechanism may underlie EMT in EC.

Research studies about the role of circRNA in EC are still in the early phase. Thus, circRNA-seq enables a better understanding of the features associated with the overall expression of circRNAs in EC. Firstly, circRNAs are a special class of small non-coding RNAs, and a panoramic view of circRNAs in EC should be compared with that of other types of small non-coding RNAs such as miRNAs and PIWI-interacting RNAs (piRNAs). Secondly, circRNAs-seq indicated that circRNAs may play an important role in EC. A major limitation of the aforementioned studies was that the sample size of clinical EC patients was relatively small. Future studies should include larger EC samples to assess the role of circRNAs at different stages and subtypes of EC. In addition, it would be interesting to compare circRNA expression in endometrial hyperplasia with or without cytological atypia. Endometrial hyperplasia with cytological atypia indicates a tendency toward cancer (12–14). Thus, differentially expressed circRNAs may be useful for obtaining a better understanding of EC.

### Individual circRNAs in EC

Regarding the role of individual circRNAs in EC, the expression level of circ_PUM1 was significantly higher in EC tissues than in normal tissues (15). Circ_PUM1 overexpression promoted the proliferation, metastasis, and invasion of EC cells. In contrast, the ability of EC cells to grow into tumors was reduced following circ_PUM1 knockdown. Mechanistically, circ_PUM1 bound with miR-136, which targeted Notch homolog 3 (NOTCH3, an identified oncogene of EC). Sponging of miR-136 by Circ_PUM1 lead to activation of NOTCH3, thereby promoting EC development.

EC patients displaying high expression levels of circ_0002577 showed poor overall survival and advanced tumor stages (16). Overexpression of circ_002577 in EC cells promoted their proliferation, migration and invasion, whereas circ_002577 knockdown in EC cells demonstrated the opposite effect. Furthermore, circ_002577 accelerated EC progression by sponging miR-625-5p and upregulating insulin like growth factor 1 receptor (IGF1R) (17). Another recent study validated the oncogenic role of circ_0002577 in EC *via* miR-197/catenin delta 1(CTNND1)/Wnt axis, suggesting that circ_002577 is a potentially therapeutic factor in EC.

CircWHSC1 overexpression promoted the proliferation, migration, and invasion of EC cells while decreasing the apoptosis of these cells (18). EC-derived tumors overexpressing CircWHSC1 showed increased tumorigenicity as indicated by a study investigating nude mouse xenograft tumor models. Reportedly, circWHSC1 binds to miR-646 and induces the expression of nucleophosmin 1 (NPM1), a downstream target of miR-646, in EC cells. This suggests that CircWHSC1 promotes EC development by sponging miR-646 and targeting NPM1.

The expression of circTNFRSF21 was up-regulated in EC tumor tissues (19). circTNFRSF21 promoted EC cell growth *in vitro* and tumor formation *in vivo*. Mechanically, circTNFRSF21 acted as a sponge of miR-1227 to restore mitogen-activated protein kinase 13 (MAPK13)/activating transcription factor 2(ATF2) signaling pathway activity in EC cells. Hsa_circ_0061140 is another oncognetic circRNA in EC cells (20). Hsa_circ_0061140 acted as a sponge for miR-149-5p, which was proven as a tumor suppressor in tumor growth and metastasis (21). Signal transducer and activator of transcription 3 (STAT3) was proved as another target of miR-149-5p. Moreover, hsa_circ_0061140 exhibited its oncogenic role due to the modulation of miR-149-5p/STAT3 axis.

**Figure 1:**
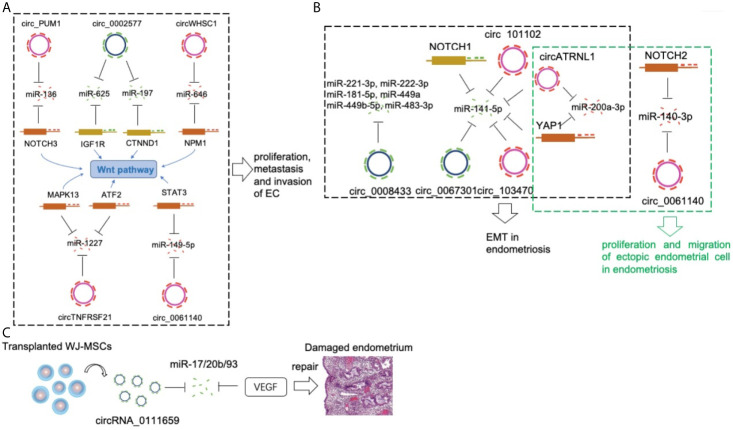
CircRNAs in a pathological endometrium. **(A)** Circ_PUM1, circ_0002577, circWHSC1, circTNFRSF21 and circ_0061140 compete with NOTCH3, IGF1R, NPM, MAPK13/ATF2 and STAT3 *via* sponging miR-136, miR-625/miR-197, miR-646, miR-1227 and miR-149-5p respectively, to promote EC development. **(B)** The circ_0067301/miR-141e-5p/NOTCH1, circ_103470/miR-141-5p, circ_101102/miR-141-5p, circATRNL1/miR-141-5p/miR-200a-3p/YAP1, circ_0061140/miR-140-3p/NOTCH2 axis played an important regulatory role in the EMT and proliferation/migration in endometriosis. **(C)** The circRNA_0111659-miR-17/20b/93-VEGF association is involved in regulating endometrial damage repair.

The number of studies of individual circRNA in EC is limited. There are only six related reports up to now. Among them, there are two independent studies that focus on a common circRNA, circ_0002577. Moreover, all these reported circRNAs involved in two basic functional processes of EC cells, proliferation and migration. Interestingly, Wnt/β-Catenin pathway has been proven as a central signaling pathway to connect the abovementioned individual circRNAs (**Figure 1A**). The interaction of circRNAs and Wnt pathway in EC needs further investigation.

## circRNAs in Endometriosis

Endometriosis is a common gynecological disease in women, which is caused by active endometrial cells that are present outside the endometrium. Endometrial cells are naturally intended to grow in the uterine cavity. The uterine cavity is connected to the pelvic cavity *via* the fallopian tube. However, endometrial cells may grow ectopically by moving through the fallopian tube. Many theories have been proposed to explain the pathological mechanism underlying this disease (22). Emerging evidence suggests that many factors, including immune cells, adhesion molecules, extracellular matrix metalloproteinases and pro-inflammatory cytokines shape the environment for survival, adhesion and differentiation of ectopic endometrial cells (23, 24). The disease mostly occurs in women of childbearing age. Ectopic lesions may gradually shrink and degenerate during the postmenopausal period (25).

### High-Throughput Studies of circRNAs in Endometriosis

Six pairs of ectopic and eutopic endometria were subjected to high-throughput RNA-seq to analyze the expression patterns of circRNAs in endometriosis, which was followed by qRT-PCR analysis of 30 pairs of samples (26). The analyses identified 146 upregulated and 148 downregulated circRNAs. Based on 2,495 MREs, a ceRNA network consisting of 48 miRNAs and 296 mRNAs was identified. GO functional analysis and KEGG pathway analysis revealed several circRNA-associated signaling pathways, such as MAPK, PI3K-AKT and FOXO, which may be associated with the pathogenesis of endometriosis. Abnormal activation of MAPK pathway in primary eutopic endometrial stromal cells of patients with endometriosis induced cell proliferation and migration (27). The communications between these pathways and circRNAs may provide a new direction for the prevention, diagnosis and treatment of endometriosis (28).

Microarray assays were performed on endometrial tissues from patients with endometriosis and normal endometrial tissues, followed by qRT-PCR validation, which identified 262 upregulated and 291 downregulated circRNAs, binding to 1,225 MREs (29). The ceRNA networks included 122 miRNAs and 137 mRNAs, which were closely related to nine signaling pathways. Some of them, such as Rap1 pathway (30) and leukocyte transendothelial migration pathway(including Intercellular Adhesion Molecule 1[ICAM-1) and Integrin Subunit Alpha L (ITGAL)] (31, 32), have been proven as involved in endometriosis. The data suggest that these pathways might play a role in the pathogenesis and development of endometriosis *via* interacting with circRNAs.

Researchers analyzed the expression patterns of circRNAs in ectopic and paired eutopic endometria and constructed a circRNA-miRNA-mRNA network (33). Expression-related characteristics of circRNA and mRNA in four patients were evaluated using microarray analysis. Eight circRNAs and mRNAs in 37 patients were validated using qPCR. In addition, 1,258 upregulated and 1,061 downregulated circRNAs, as well as 1,900 upregulated and 2,535 downregulated mRNAs, were detected between ectopic and control endometria. Functional analysis showed that most of the differentially expressed mRNAs were involved in immune-inflammatory responses and cell-cycle regulation. Five circRNAs (circ_0004712, circ_0002198, circ_0003570, circ_0008951, and circ_0017248) and eight mRNAs (SCN3B, ENTPD1, IL16, BACH2, C3, CKS2, G0S2, and PGRMC1) identified using qPCR matched those shown in microarray results. In addition, a circRNA-miRNA-mRNA network was constructed, revealing the participation of cancer-related purine metabolism, glyceride metabolism, and thyroid hormone signaling pathways in the pathogenesis of endometriosis. The results of this study indicated that circRNAs have potential as promising diagnostic biomarkers of ovarian endometriosis as well as therapeutic targets for this disease.

Another study included 63 clinical samples (control endometrium, *n* = 22; and ectopic endometrium, *n* = 41) (34). Of these, four samples in each group were used for circRNA microarray assays. Of the 88 significantly changed circRNAs, 11 and 77 circRNAs were upregulated and downregulated, respectively, in the ectopic endometrium of endometriosis patients. The qRT-PCR validation results of two induced circRNAs (circ_0004712 and circ_0002198) matched microarray assay results. These two circRNAs may represent potential new biomarkers useful for diagnosing endometriosis.

Similar to EC, research of circRNA in endometriosis also focused on the illumination of a “circRNAome” *via* sequencing techniques. Mechanisms underlying the alteration of physiological functions caused by circRNAs in endometriosis need to be further elucidated. This will broaden application of the results, as these may function not only as potential targets for treatment of endometriosis but also as diagnostic biomarkers of the disease. In addition, studies based on *in vitro* experiments as well as *in vivo* animal models, such as primate models and patient-derived xenograft models (PDX) (35, 36), should be performed to further clarify the pathophysiological relevance of circRNAs in endometriosis.

### Individual circRNAs in Endometriosis

Epithelial-mesenchymal transition (EMT) is a core pathological mechanism associated with endometriosis (37, 38). A study was designed to investigate the role of circRNAs and miRNAs during the EMT process in endometriosis (39). Compared with those in control endometria, the expression levels of hsa_circ_0067301 and miR-141e-5p in the ectopic endometria were significantly reduced. Hsa_circ_0067301 knockdown promoted the proliferation and migration of Ishikawa cells and End1/E6E7 cells. Some EMT-related markers, such as Notch-1, Hes-1, N-cadherin, and vimentin, were upregulated, whereas E-cadherin was downregulated. EMT was partially relieved following co-infection with miR-141e-5p inhibitors. These results showed that the hsa_circ_0067301/miR-141e-5p/Notch-1 pathway plays an important regulatory role in EMT during endometriosis.

Another study included four patients with endometriosis and four with general endometritis (without endometrial disease) (40). Patients with endometriosis provided ectopic endometrial tissues and paired normal endometrial tissues. This study identified 2,233 differentially expressed circRNAs among these three groups. In addition, eight circRNAs were closely associated with EMT, which is a prerequisite for the establishment of endometriotic lesions. The results also showed that two circRNAs (circ_103470 and circ_101102) regulated EMT in endometriosis by sponging miR-141-5p, which may represent a promising target for the treatment of endometriosis (41). Interestingly, miR-141-5p is a common EMT-related miRNA in both studies. These studies highlighted the importance of circRNAs in the process of EMT in endometriosis and provides unique insights into the molecular basis of the pathogenesis of endometriosis (**Figure 1B**).

Circ_0061140 only acts as oncogenic regulator in EC (20), it also plays a role in endometriosis (42). Knockdown of circ_0061140 inhibited proliferation and migration of ectopic endometrial cell. MiR-140-3p decreased in ectopic endometrial cells and it acted as a direct target of circ_0061140 by repressing the effects of circ_0061140 on ectopic endometrial cells. A positive correlation between circ_0061140 and Notch homolog 2 (Notch2) was demonstrated in endometriosis. Moreover, circ_0061140 could induce endometriosis progression *via* miR-140-3p-Notch2 axis.

Knockdown of Circ_0008433 repressed proliferation, migration and angiogenesis and induced apoptosis of endometrial stromal cells (43). Six potential target miRNAs (miR-221-3p, miR-222-3p, miR-181-5p, miR-449a, miR-449b-5p, miR-483-3p) were significantly changed in circ_0008433-overexpressing endometrial stromal cells. Furthermore, Protein-protein interaction (PPI) analysis showed that up-regulation of circ_0008433 modulated EMT in endometriosis through the circRNA–miRNA–mRNA axis.

CircATRNL1 and Yes-associated protein 1 (YAP1) increased and miR-141-3p and miR-200a-3p decreased in ectopic tissues (44). CircATRNL1 and YAP1 induced the proliferation and migration and induce EMT by targeting miR-141-3p and miR-200a-3p in endometrial cells. Rescue assays further validated the role of circATRNL1–miR-141-3p/miR-200a-3p–YAP1 axis in endometrial cells, which could contribute to the progression of endometriosis.

There are five studies of individual circRNA in endometriosis until now. Interestingly, four circRNAs (circ_0067301, circ_103470, circ_101102, and circATRNL1) promoted EMT in endometriosis by targeting a common miRNA, miR-141. The essential inhibitory effects of miR-141 on proliferation/migration (45) and EMT (46) also have been proven in endometrial cells. In addition, two circRNAs (circ_0067301 and circ_0061140) induced the expression of different members of the Notch family, Notch1 and Notch2 in endometriosis. Given that the hyperactivation of Notch family (47) and the effects of Notch pathway on EMT (48) have been reported in endometriosis, the role of circRNA-miRNA-Notch axis in EMT of endometriosis definitely should be deeply investigated in the future.

## circRNAs in Other Endometrial Diseases

Other endometrial-related disorders, such as endometrial damage and implantation failure, also greatly affect the quality of life of women (49). Wharton’s jelly derived mesenchymal stem cells (WJ-MSCs) have shown great potential for repairing different diseases. Human endometrial stromal cells (ESCs) damaged by culturing with mifepristone were repaired by WJ-MSCs (50). The repairing of damaged ESCs in the co-culture group by WJ-MSCs, improved cell morphology, increased proliferation and decreased apoptosis. The circRNA microarray analyses indicated that 7,757 circRNAs showed differential expression in ESCs co-cultured with WJ-MSCs. In addition, researchers focused on hsa_circRNA_0111659 and predicted it to be related to miR-17/20b/93 and the target mRNA, VEGF. The circRNA-miRNA-mRNA combination may be involved in regulating endometrial damage repair. The results showed that abundant circRNAs were expressed during repair of damaged endometria by WJ-MSC, providing a new perspective in regard to the mechanism of endometrial repair by WJ-MSCs (**Figure 1C**).

The specific role of circRNAs in the pathogenesis of repeated implantation failure remains unclear (51). CircRNA expression in the endometrial biopsies of six women showing repeated implantation failure and a control group (six healthy women) was screened using microarrays (52). Data from this circRNA microarray assay showed that 856 unique circRNAs were significantly altered. Subsequently, qRT-PCR verified the upregulation of hsa_circRNA_070616, hsa_circRNA_103716, hsa_circRNA_104001, and hsa_circRNA_104854 and the downregulation of hsa_circRNA_ 004183, hsa_circRNA_044353, and hsa_circRNA_404686. Differentially expressed circRNAs provided new target molecular candidates for the diagnosis and clinical treatment of patients with repeated implantation failures.

Endometrial receptivity is defined as the ability of the endometrium to accept an embryo implantation (53, 54). Studies directed at the potential molecular mechanisms that may be involved, centered on protein-coding genes in the field of assisted reproduction (55). In addition, circRNA microarray assays, used to compare circRNA expression in the early secretory phase and mid-secretory phase endometrium, identified a number of circRNAs (hsa_circRNA_101280, hsa_circRNA_102293, hsa_circRNA_104789, hsa_circRNA_104791, hsa_circRNA_101263, hsa_circRNA_103493, hsa_circRNA_104625, has_circRNA_400019 and hsa_circRNA_104700), which may be useful as potential biomarkers of endometrial receptivity (56). Adenomyosis is a benign uterine disorder characterized by presence of endometrium in the myometrium (57). The down-regulation of hsa_circRNA_101280 was validated in adenomyosis samples (n=11) compared with that in the control samples (n=11), suggesting a potential mechanism underlying decreased implantation rates observed in women with adenomyosis (56). This study not only expands the knowledge of circRNAs in human endometrium but also provides useful clues for understanding the role of circRNAs in endometrial receptivity.

Researchers used Illumina Solexa techniques to analyze circRNAs in the endometria of three goats at gestational day 5 (pre-receptive endometrium, PE) and three goats at gestational day 15 (receptive endometrium, RE) (58). Overall, 21,813 circRNAs were identified, of which 5,925 circRNAs were RE- and 9,078 circRNAs were PE-specific, indicating the high stage-specificity of circRNAs. Further analyses indicated that there were 334 differentially expressed circRNAs in the RE stage compared with those in the PE stage. Analyses of the cyclic RNA-miRNA interaction network further supported the contention that circRNAs may be used as miRNA sponges to regulate gene expression. In addition, estrogen/progesterone regulated some circRNAs in the endometrial epithelial cells (EECs) and ESCs. These data were used to compile a circRNA atlas of the goat endometrium during embryo implantation.

CircRNA-miRNA-mRNA networks were constructed to explore the involvement of ceRNA in the development of RE in goats (59). Cyclic RNA8073 (ciR-8073) reduced miR-181a levels in a manner similar to that of a miRNA sponge. This effect indirectly increased the expression of neurotensin in EECs. Neurotensin promotes BCL-2 expression *via* the MAPK pathway and induces expression of leukemia inhibitor factor, cyclooxygenase 2 (COX-2), vascular endothelial growth factor A (VEGFA), and homeobox A10 (HOXA10). This indicated the presence of a ciR-8073-miR181a-neurotensin pathway in the endometrium of goats. CiR-8073 functions as a ceRNA sequestering miR-181a, thereby protecting neurotensin from miR-181a-mediated suppression in EECs.

**Figure 2:**
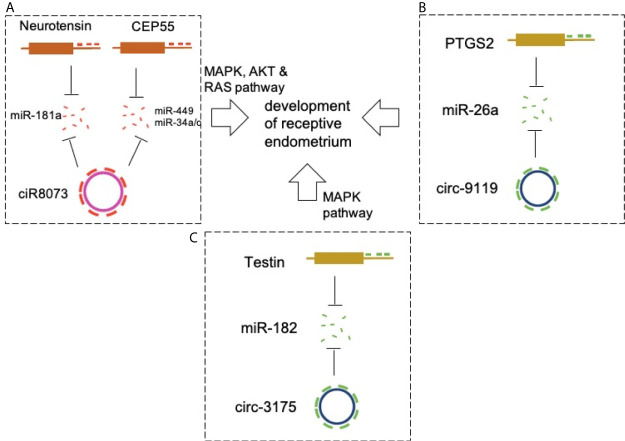
CircRNAs in endometrial receptivity. **(A)** CiR-8073 functions as a ceRNA to sequester miR-181a, thereby protecting neurotensin from miR-181a-mediated suppression in EECs. Circ-8073 regulates endometrial receptivity *via* miR-181a/neurotensin and miR-449a/CEP55 and miR-34/CEP55 axes. **(B)** Modulation of the circRNA-9119-miR-26a-PTGS2 axis in EECs may be a potential target for regulating RE development. **(C)** The ciR3175-miR182-testin axis is essential for development of pre-receptive endometrium in goats.

Circ-8073 directly binds miR-449a and inhibits its activity (60). Centrosomal protein 55 (CEP55) is a direct target of miR-449a. Circ-8073 improves the expression of CEP55 by absorbing miR-449a in EECs *in vitro*. Circ-8073/miR-449a/CEP55 promotes EC proliferation *via* the PI3K/AKT/mTOR pathway. In addition, CEP55 regulates the expression of VEGF and FOXM1 in EECs, thereby contributing to the formation of endometrial receptors. These findings in goats suggest that circ-8073 regulates endometrial receptivity *via* miR-449a/CEP55 and PI3K/AKT/mTOR pathways. Another study also validated the promotive effects of circ-8073 on EEC proliferation *via* competitive sponging of miR-34a/c *via* CEP55 (61) (**Figure 2A**).

A similar study detected high expression of circRNA-9119 and prostaglandin-endoperoxide synthase 2 (PTGS2) and low levels of miR-26a at the RE stage of goats (62). Further studies showed that circRNA-9119 reduces miR-26a by acting as a miRNA sponge, and it is known that miR-26a reduces the expression of PTGS2 in goat EECs. In addition, PTGS2 is involved in the regulation of certain protein markers of endometrial receptivity in goat EECs. Therefore, modulation of the circRNA-9119-miR-26a-PTGS2 pathway in EECs may be a potential target in the regulation of RE development (**Figure 2B**).

The levels of circRNA3175 (ciR3175) and testin in the goat pre-receptive endometrium were high, whereas the expression level of miR-182 was low (63). Further studies showed that ciR3175 and testin functioned as ceRNAs by competitively sponging miR-182 in EECs. In addition, testin inhibited EEC apoptosis by reducing the expression levels of BCL-2/BAX *via* the MAPK pathway. Therefore, the ciR3175-miR182-testin may be essential for the development of pre-receptive endometrium in goats. High-quality circRNA expression profiles were obtained from endometrial tissue in these studies (**Figure 2C**).

A better understanding of the conditions associated with endometrial receptivity may help in improving the rate of embryonic bedding, which positively affects the treatment of female infertility (64). Mechanistic research has concentrated on ceRNA regulatory networks, such as the ciR-8073-miR181a-neurotensin, circ-8073/miR-449a/miR-34/CEP55, circRNA-9119-miR-26a-PTGS2, and ciR3175-miR182-testin networks. Many circRNAs are differentially expressed between PE and RE. Following GO and KEGG pathway analysis, interaction network analysis of circRNA-miRNA-mRNA and analysis of circRNAs and their host genes may improve our understanding of how circRNAs mediate the regulation of target genes in the development of endometrial receptivity. The above studies have demonstrated that some circRNAs exert their biological effects on endometrial receptivity by competitively sponging miRNA, thus inducing the expression of miRNA’s targets. In the studies of ciR8073, this circRNA was validated as target for three miRNAs (miR-181a, miR-449 and miR-34a/c), suggesting that a circRNA was regulated by multiple miRNAs, and further analysis should investigate whether a miRNA could be targeted for multiple circRNAs in endometrial receptivity. However, in addition to ceRNA regulatory mechanisms, other underlying mechanisms, such as circRNA-protein interactions that also lead to significant changes in circRNA expression as well as the function of individual circRNAs in endometria, need further investigation. These findings will expectedly increase the diversity of the endometrial transcriptome for circRNAs and yield new insights into the development of endometrial receptivity.

## Conclusion and Future Direction of circRNAs in the Endometrium

Thousands of circRNAs are encoded by the human genome in a context-dependent manner (65). Due to their abundant expression, stability, and multiple MREs, a large number of circRNAs function *via* ceRNA regulatory mechanisms (66). circRNAs interact with RNA-binding proteins *via* direct binding, which constitutes another major regulatory mechanism of circRNAs (67). In addition, inflammation of endometrium is a complex condition, which is almost involved in all endometrial disorders (68–70). Further research is needed for a better understanding of circRNAs in inflammation of reproductive tract.

Certain properties of circRNAs, such as small size and stability, are ideally suited for a role as biomarkers. Furthermore, tissue-specific expression patterns of circRNAs are closely associated with clinical phenotypes. In addition, circRNAs, which are not affected by endonuclease degradation and are stable in formalin-fixed paraffin-embedded tissues (71), can be detected in vaginal secretions (72), and therefore should be investigated further for the purpose of diagnosing related diseases.

Due to being considered as a “hotspot” of RNA research, an increasing number of circRNAs are identified *via* effective high-throughput sequencing techniques and bioinformatics. However, studies pertaining to the emerging role of circRNAs in the endometrium are yet in the formative stages. Advanced databases, testing tools, and research techniques have enabled circRNAs to be recognized as potential non-invasive biomarkers of reproductive as well as gynecological diseases. In addition, there is also a lack of studies evaluating the panoramic view of circRNAome and individual circRNA along the entire menstrual cycle in human endometrium. Considered together, circRNA research allows researchers to enter a new level in epigenetic regulatory networks, whereby future research may lead to a better understanding of the mechanisms regulating circRNAs in the endometrium.

## Author Contributions

JT and YC (3rd author) drafted the manuscript. HY, YC (4th author), HC, ZL, LL, YZ, XC, and ZY revised the article. All authors contributed to the article and approved the submitted version.

## Funding

This study was supported by grants from the Sanming Project of Medicine in Shenzhen (SZSM201812041) and Clinical Research Funding from Shenzhen Second People’s Hospital (4001023).

## Conflict of Interest

The authors declare that the research was conducted in the absence of any commercial or financial relationships that could be construed as a potential conflict of interest.
